# Inpatient Stress Test Modality and Downstream Clinical Pathways in Troponin-Negative Patients: A Real-World Cohort Study

**DOI:** 10.7759/cureus.108444

**Published:** 2026-05-07

**Authors:** Admire Hlupeni, Sudden Turugari, Jeffrey Murray

**Affiliations:** 1 Immunology, Midlands State University, Gweru, ZWE; 2 Internal Medicine, St. Luke's Hospital, Chesterfield, USA; 3 Internal Medicine, Midlands State University, Gweru, ZWE; 4 Cardiology, St. Luke's Hospital, Chesterfield, USA

**Keywords:** clinical pathways, coronary artery disease, invasive coronary angiography, myocardial perfusion imaging, stress echocardiography, stress testing, troponin-negative

## Abstract

Background

Troponin-negative patients presenting with suspected acute coronary syndrome are frequently admitted for inpatient stress testing. The relative yield of different stress test modalities and their influence on downstream clinical decision-making in this population remain uncertain.

Methods

We conducted a retrospective cohort study of 769 consecutive troponin-negative adult patients (≥18 years) undergoing inpatient stress testing at St Luke’s, a community teaching hospital in the Midwest, USA. Stress test modalities were categorized as nuclear myocardial perfusion imaging (MPI), stress echocardiography, or exercise electrocardiography (ECG)-only. The primary outcome was stress test positivity defined as the presence of inducible ischemia as interpreted by the reading cardiologist. This included reversible perfusion defects on nuclear MPI, inducible regional wall motion abnormalities on stress echocardiography, or ischemic ECG changes during exercise testing. Secondary outcomes included referral to invasive coronary angiography (ICA), detection of obstructive coronary artery disease (CAD), and subsequent revascularization. Multivariable Poisson regression models with robust standard errors were used to evaluate the association between stress test modality and outcomes after adjustment for baseline characteristics.

Results

Nuclear MPI was the most commonly conducted modality (n=616, 80.1%), followed by stress echocardiography (n=144, 18.7%) and exercise ECG-only testing (n=9, 1.2%). Comparisons were performed between nuclear MPI and stress echocardiography, as the ECG-only subgroup was small. Stress test positivity rates were similar between nuclear MPI and stress echocardiography (n=60, 9.7% vs n=12, 8.3%, p=0.80). No positive tests were observed in the ECG-only group, though this subgroup was very small. Referral to ICA was primarily driven by stress test results rather than modality. Among patients with positive stress tests, ICA was performed in 80% (48/60) of nuclear MPI and 75% (9/12) of stress echocardiography cases (p=0.71), whereas ICA was infrequently performed following negative tests (5.8% (32/556) vs 2.3% (3/132), respectively; p=0.23). Overall, ICA utilization did not differ significantly between nuclear MPI (n=80, 13.0%) and stress echocardiography (n=12, 8.3%), p=0.21. Among patients undergoing ICA, rates of obstructive CAD (37.5% (30/80) vs 41.7% (5/12), p=0.76) and revascularization (32.5% (26/80) vs 41.7% (5/12), p=0.53) were also similar between nuclear MPI and stress echocardiography groups, respectively. In adjusted analyses, stress test modality was not independently associated with stress test positivity (adjusted risk ratio 0.97, 95% CI 0.51-1.85; p=0.94) or referral to ICA (adjusted risk ratio 0.61, 95% CI 0.32-1.14; p=0.12).

Conclusions

In this single-center retrospective cohort, both unadjusted and adjusted analyses demonstrated similar stress test yield and downstream referral to ICA between nuclear MPI and stress echocardiography. These findings suggest that, in this setting, stress test modality may have limited influence on clinical decision-making following negative troponin evaluation. Despite consistent findings after adjustment, residual confounding cannot be excluded; therefore, these results should be interpreted as observational associations rather than evidence of causal effects.

## Introduction

In patients presenting with suspected acute coronary syndrome (ACS) in the emergency department (ED), serial cardiac troponin testing is central to excluding myocardial infarction (MI) [1-3]. Despite negative troponins, many patients undergo inpatient stress testing to evaluate for inducible ischemia, a practice endorsed as reasonable by AHA/ACC guidelines for patients with possible ACS who have normal serial ECGs and troponins [4,5]. Multiple stress testing modalities are available, including nuclear myocardial perfusion imaging (MPI), stress echocardiography, and exercise electrocardiography (ECG)-only testing [6,7].

Although chest pain evaluation has evolved with high-sensitivity troponin assays and structured risk stratification pathways, inpatient stress testing remains commonly performed in many real-world settings despite negative troponin evaluation. While these modalities differ in their technical characteristics and reported diagnostic performance under controlled conditions [8], their real-world impact on downstream clinical decision-making, particularly in troponin-negative patients, is less well understood. In practice, stress testing functions as a gatekeeper to downstream invasive evaluation, and whether differences in modality translate into differences in clinical pathways remains uncertain [9].

We therefore sought to determine if stress test modality is associated with differences in real-world clinical pathways in troponin-negative patients undergoing inpatient evaluation. Specifically, we examined differences in stress test positivity, referral to invasive coronary angiography (ICA), detection of obstructive coronary artery disease (CAD), and revascularization across commonly used stress test modalities.

## Materials and methods

Study design

This was a retrospective cohort study of consecutive adult patients (≥18 years) presenting to the ED with suspected myocardial ischemia at St. Luke’s Hospital, a large community teaching hospital in the Midwest, USA.

Patient identification and selection

Patients were identified through an institutional electronic health record query of all inpatient stress tests performed between May 2024 and May 2025. During this period, conventional (non-high-sensitivity) troponin assays were used, as high-sensitivity assays had not yet been implemented at the institution. Potentially eligible patients were those who presented to the ED with symptoms suggestive of MI (such as chest pain, dyspnea, or equivalent symptoms), underwent serial cardiac troponin I testing, and were admitted for inpatient stress testing during the index hospitalization at the discretion of the ED physician or consulting cardiologist.

The study included adult patients with negative serial troponins. Serial troponin testing was defined as ≥2 measurements obtained at 0, 2, and 6 hours, per institutional protocol. A positive troponin value was defined as >0.12 ng/mL. Patients with ST-elevation myocardial infarction, active arrhythmias or any positive troponin value were excluded.

Thus, the final cohort represents a clinically selected population of troponin-negative patients admitted for further ischemia evaluation with inpatient stress testing.

Baseline risk: modified HEART score 

A modified history, electrocardiogram, age, risk factors, and troponin (HEART) score was retrospectively calculated for all included patients. To reduce subjectivity inherent in retrospective chart review, the history component was standardized and assigned two points for every patient, reflecting that all individuals had symptoms considered concerning enough to justify hospital admission and inpatient stress testing. The remaining components (age, ECG, and risk factors) were calculated according to established HEART score criteria [10]. Given that all patients met inclusion criteria of negative serial troponins, the troponin component was uniformly assigned 0 points. In keeping with HEART score definitions, obesity was defined as a body mass index (BMI) >30 kg/m². Patients were subsequently categorized into low-risk (0-3), intermediate-risk (4-6), and high-risk (7-10) groups.

Stress test modalities

Stress test modalities were categorized into three groups: (i) Nuclear MPI, including regadenoson or exercise single-photon emission computed tomography (SPECT), (ii) Stress echocardiography (exercise or pharmacologic [dobutamine]), and (iii) Exercise electrocardiography (ECG)-only testing without imaging

Given the small sample size, the ECG-only group was included for descriptive purposes but not emphasized in comparative analyses.

Outcomes

The primary outcome was stress test positivity, defined as inducible ischemia as interpreted by the reading cardiologist. Secondary outcomes included referral to ICA during the index hospitalization or within 90 days of the stress test, presence of obstructive CAD (≥50% stenosis of the left main coronary artery or ≥70% of a major epicardial vessel) on ICA and coronary revascularization (percutaneous coronary intervention or coronary artery bypass grafting).

Statistical analysis

Continuous variables are presented as means with standard deviations, and categorical variables are summarized as frequencies and percentages. Comparisons across stress test modalities were performed using Student’s t-test for continuous variables and Fisher’s exact test for categorical variables, given the expected low frequency of outcomes in certain subgroups.

To account for baseline differences between groups, multivariable Poisson regression models with robust standard errors were constructed to evaluate the association between stress test modality, and stress test positivity and referral to ICA. Covariates were selected a priori based on clinical relevance and included age, sex, cardiovascular risk factors, and established atherosclerotic cardiovascular disease (ASCVD).

Referral to ICA was not protocol-driven and reflected clinician judgment by the managing clinicians, introducing potential confounding by indication. Analyses of outcomes among patients undergoing ICA were considered exploratory and should be interpreted with caution due to potential verification bias. All analyses were performed using Stata version 19.5 (StataCorp, College Station, TX).

## Results

Study population and baseline characteristics

A total of 769 patients were included in the analysis. Nuclear MPI was the predominant modality (n=616, 80.1%), followed by stress echocardiography (n=144, 18.7%) and exercise ECG-only testing (n=9, 1.2%) (Table 1).

**Table 1:** Baseline patient characteristics by stress test modality (N= 769). Categorical variables are presented as n (%), and continuous variables as mean +/- standard deviation. Percentages represent the distribution of baseline characteristics within the overall cohort and stratified by stress test modality. Nuclear myocardial perfusion imaging (MPI) included both exercise and pharmacologic (regadenoson (Lexiscan)) protocols, while stress echocardiography included both exercise and dobutamine protocols. Unless otherwise specified, p-values were calculated using Fisher’s exact test to compare nuclear myocardial perfusion imaging (MPI) and stress echocardiography groups. The exercise electrocardiogram (EKG)-only group was excluded from statistical comparisons due to its small sample size. ^£^p-values were calculated using the Student’s t-test for continuous variables. ^#^subset of coronary artery disease. ASCVD: atherosclerotic cardiovascular disease; BMI: body mass index; CABG: coronary artery bypass grafting; CAD: coronary artery disease; ECHO: echocardiography; EKG: electrocardiogram; ESRD: end-stage renal disease; MPI: myocardial perfusion imaging; PCI: percutaneous coronary intervention; SD: standard deviation; ST: stress test.

Characteristic		Total Cohort (N=769)	Nuclear MPI (n=616)	Stress ECHO (n=144)	Exercise EKG-only (n=9)	p-value
Baseline Demographics						
Male sex		357 (46.4)	284 (46.1)	70 (48.6)	3 (33.3)	0.64
Mean Age +/- SD, years		68.6 +/- 12.5	70.5 +/- 11.7	61.7 +/- 12.1	50.7 +/- 13.3	<0.0001^£^
Mean BMI +/- SD, kg/m^2^		30.6 +/- 6.8	30.9 +/- 7.0	29.3 +/- 5.9	30.1 +/- 5.0	0.01^£^
Chief Presenting Symptom						0.09
	Chest Pain	572 (74.4)	449 (72.9)	117 (81.3)	6 (66.7)	
	Dyspnea	80 (10.4)	70 (11.4)	9 (6.3)	1 (11.1)	
	Other	117 (15.2)	97 (15.8)	18 (12.5)	2 (22.2)	
Cardiovascular Risk Factors						
Hypertension		533 (69.3)	453 (73.5)	75 (52.1)	5 (55.6)	<0.0001
Diabetes Mellitus		183 (23.8)	163 (26.5)	20 (13.9)	0 (0)	0.001
Hyperlipidemia		501 (65.2)	429 (69.6)	71 (49.3)	1 (11.1)	<0.0001
Current smoking		66 (8.6)	53 (8.6)	13 (9.0)	0 (0)	0.87
Established ASCVD		281 (36.5)	252 (40.9)	29 (20.1)	0 (0)	<0.0001
	CAD	265 (34.5)	237 (38.5)	28 (19.4)	0 (0)	<0.0001
	Prior CABG/PCI^#^	222 (28.9)	198 (32.1)	24 (16.7)	0 (0)	<0.0001
Family History of CAD		431 (56.1)	353 (57.3)	76 (52.8)	2 (22.2)	0.35
ESRD on dialysis		11 (1.4)	11 (1.8)	0 (0)	0 (0)	0.24
Rheumatoid Arthritis		42 (5.5)	34 (5.5)	8 (5.6)	0 (0)	1
Other Comorbidities						
Heart failure		77 (10.0)	75 (12.2)	2 (1.4)	0 (0)	<0.0001
Atrial fibrillation/ flutter		141 (18.3)	131 (21.3)	10 (6.9)	0 (0)	<0.0001
Clinical Scoring						
Modified HEART Score						<0.0001
	Low risk (0-3)	26 (3.4)	12 (2.0)	11 (7.6)	3 (33.3)	
	Moderate risk (4-6)	681 (88.6)	547 (88.8)	128 (88.9)	6 (66.7)	
	High risk (7-10)	62 (8.1)	57 (9.3)	5 (3.5)	0 (0)	

Compared to those who underwent stress echocardiography, patients undergoing nuclear MPI were older and had a higher burden of cardiovascular comorbidities, including hypertension, diabetes mellitus, hyperlipidemia, and established ASCVD. The distribution of chief presenting symptoms was similar across groups, with chest pain being the most common presenting complaint. Modified HEART score categories differed significantly between modalities, reflecting variation in baseline risk profiles. These findings suggest that stress test modality selection was influenced by patient characteristics and clinical risk assessment at presentation.

Stress test positivity

**Figure 1:**
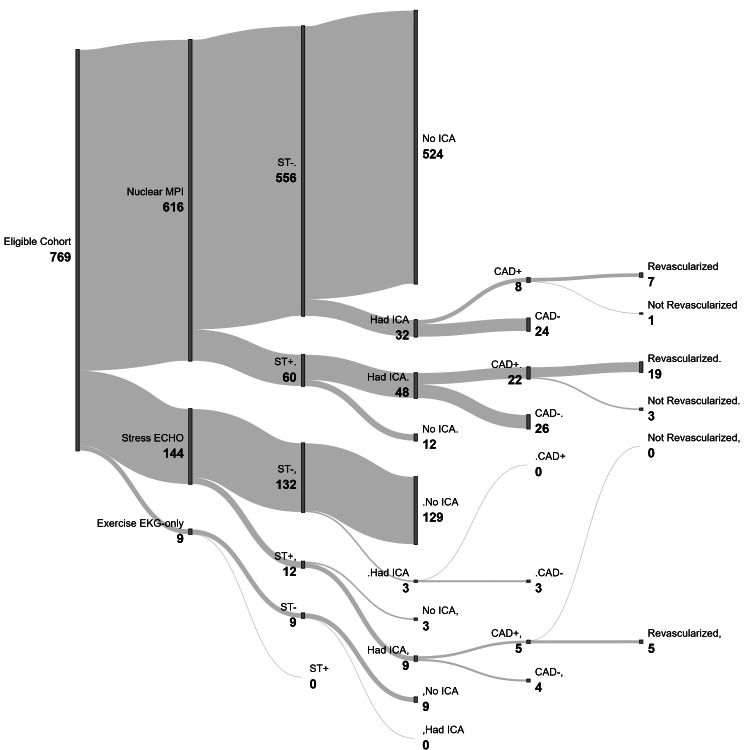
Downstream diagnostic pathways following inpatient stress testing by modality. The figure illustrates patient flow from stress test modality (nuclear myocardial perfusion imaging (MPI), stress echocardiography, and exercise electrocardiography (ECG)-only) to stress test result (positive vs negative), subsequent referral to invasive coronary angiography (ICA), detection of obstructive coronary artery disease (CAD), and revascularization. The width of each flow is proportional to the number of patients at each step. The diagram demonstrates that downstream clinical decision-making is primarily driven by stress test results rather than modality. CAD+: presence of obstructive coronary artery disease; CAD-: no obstructive coronary artery disease; CAD: coronary artery disease; ECHO: echocardiography; EKG: electrocardiogram; ICA: invasive coronary angiography; MPI: myocardial perfusion imaging; ST: stress test; ST+: stress test positive; ST-: stress test negative.

Overall, 72 patients (9.4%) had a positive inpatient stress test. Stress test positivity rates were similar between nuclear MPI and stress echocardiography (9.7% (60/616) vs 8.3% (12/144), p=0.80). No positive tests were observed among patients undergoing exercise ECG-only testing; however, this subgroup was small (Figure 1).

Downstream clinical pathways

Downstream diagnostic pathways were strongly influenced by stress test results, as illustrated in Figure 1. Among patients with positive stress tests, the majority proceeded to ICA (57/72, 79%), whereas only a small proportion of patients with negative stress tests underwent further invasive evaluation (35/697, 5.0%). ICA performed following negative stress tests likely reflects persistent clinical concern on the part of the consulting cardiologist or high-risk features not fully captured by stress test results alone.

Between modalities, similar patterns were observed, with stress test results rather than modality driving referral to ICA. Overall ICA utilization did not differ significantly between nuclear MPI and stress echocardiography (13.0% (80/616) vs 8.3% (12/144); p=0.21). When stratified by stress test result, ICA was infrequently performed following negative tests (5.8% (32/556) for nuclear MPI vs 2.3% (3/132) for stress echocardiography; p=0.23) but was commonly performed following positive tests (80.0% (48/60) vs 75.0% (9/12), respectively; p=0.71).

Association between stress test modalities and outcomes

After adjustment for age, sex, BMI, chief presenting symptom, hypertension, diabetes, hyperlipidemia, established ASCVD, current smoking, atrial fibrillation, and heart failure, stress echocardiography (compared with nuclear MPI as the reference group) was not independently associated with stress test positivity (adjusted risk ratio 0.97, 95% CI 0.51-1.85; p=0.94). Similarly, stress echocardiography, compared with nuclear MPI, was not independently associated with referral to ICA after adjustment for the same baseline characteristics (adjusted risk ratio 0.61, 95% CI 0.32-1.14; p=0.12).

Obstructive coronary artery disease and revascularization

Among the 92 patients who underwent ICA, the proportion with obstructive CAD was similar between modalities (37.5% (30/80) for nuclear MPI vs 41.7% (5/12), p=0.76 for stress echocardiography; p=0.76). Rates of revascularization were also comparable (32.5% (26/80) vs 41.7% (5/12), respectively; p=0.53) (Figure 1). These findings reflect clinically selected patients and should be interpreted as exploratory.

## Discussion

In this real-world cohort of troponin-negative patients undergoing inpatient stress testing, we observed similar unadjusted rates of stress test positivity and downstream clinical pathways between modalities, despite clear differences in patient baseline characteristics. Importantly, these findings remained consistent after adjustment for baseline demographic and clinical characteristics, with stress test modality not independently associated with either stress test positivity or referral to ICA.

These findings are consistent with prior evidence demonstrating broadly comparable diagnostic performance between stress imaging modalities for the detection of obstructive CAD. Meta-analyses have shown that nuclear MPI and stress echocardiography achieve similar pooled sensitivities (approximately 81-82%) for detecting hemodynamically significant coronary stenosis, with stress echocardiography demonstrating modestly higher specificity [8,11]. The American Society of Echocardiography (ASE) guidelines similarly report comparable accuracy between these modalities, noting that meta-analyses have found similar sensitivities but higher specificity for stress echocardiography [12]. The European Association of Cardiovascular Imaging and ASE joint recommendations further affirm that noninvasive functional imaging tests demonstrate similar overall accuracy, with modality selection appropriately guided by local expertise, test availability, and patient characteristics [13]. Our findings extend this evidence by demonstrating that, in real-world inpatient practice following negative troponins, these modalities are also associated with comparable downstream clinical pathways.

In this clinical context, stress test modality appears to have limited influence on clinical decision-making. Instead, management appeared to be primarily driven by the presence or absence of a positive stress test result rather than the specific modality used. This observation is supported by a large comparative effectiveness analysis of ED patients with chest pain, which found that different noninvasive testing strategies, including stress echocardiography and myocardial perfusion scintigraphy, were associated with similar rates of downstream catheterization and revascularization, with no significant reduction in MI hospitalizations across modalities [14]. These findings underscore an important distinction between diagnostic performance under controlled conditions and its impact on real-world clinical behavior, where physician judgment and perceived patient risk often play a central role.

Importantly, baseline differences between modality groups indicate that modality selection was influenced by patient characteristics and clinical risk profile. Patients undergoing nuclear MPI were older and had a higher burden of cardiovascular comorbidities, whereas those undergoing stress echocardiography were younger and generally at lower risk. This pattern is consistent with guideline-informed practice. The 2022 ACC Expert Consensus Decision Pathway notes that stress or rest SPECT is preferred over stress echocardiography in patients who cannot exercise or who have known CAD or high coronary artery calcium burden, while stress echocardiography is favored in patients with good image quality and preserved exercise capacity [15]. Although adjusted analyses were performed, residual confounding cannot be excluded; therefore, these findings should not be interpreted as evidence of equivalence between modalities. Despite these differences in patient selection, downstream outcomes remained similar, reinforcing the observation that modality choice may not substantially alter real-world clinical pathways. This suggests that modality selection may function more as a marker of underlying risk stratification rather than a determinant of subsequent clinical decisions.

Notably, exercise ECG-only testing was infrequently performed in this cohort, likely reflecting contemporary practice patterns that favor imaging-based stress modalities. This trend is well documented. A Medicare population analysis from 2010 to 2022 demonstrated that exercise treadmill testing utilization declined from 2.3% to 1.7% of all cardiac testing, while advanced imaging modalities including PET and coronary CT angiography continued to grow [16]. The 2022 ACC Expert Consensus Decision Pathway acknowledges that exercise ECG is rarely recommended as a stand-alone test due to frequent known CAD, inability to exercise, or significant arrhythmias in the acute chest pain population [15]. Current guideline-informed approaches support the use of imaging-based stress testing in patients with baseline ECG abnormalities, reduced exercise capacity, or intermediate pretest probability, where exercise ECG alone has limited diagnostic utility [6,17]. The low utilization of ECG-only testing observed in this study is therefore consistent with evolving clinical practice and guideline recommendations.

Collectively, these findings highlight that in troponin-negative patients, the clinical utility of inpatient stress testing may lie less in the specific modality selected and more in how test results are integrated into downstream decision-making. Figure 1 further illustrates that clinical pathways converge across modalities, with stress test results serving as the primary determinant of subsequent invasive evaluation.

Study limitations

This study has several limitations. First, this was a single-center retrospective analysis that used conventional troponin assays, which may limit generalizability. Second, referral to ICA was not protocol-driven, introducing verification bias. As a result, analyses of obstructive CAD and revascularization reflect selectively referred patients and are exploratory. Third, stress test modality was not randomly assigned and differed across patient groups, introducing potential confounding by indication. Although adjusted analyses were performed, residual confounding cannot be excluded. Fourth, the exercise ECG-only subgroup was small, limiting meaningful comparisons. Finally, this study was not designed to assess intrinsic diagnostic accuracy of stress test modalities.

## Conclusions

In this single-center cohort of troponin-negative patients undergoing inpatient stress testing, stress test modality was not independently associated with differences in stress test positivity or downstream clinical pathways in both unadjusted and adjusted analyses. These findings suggest that, in this real-world setting, stress test modality may have limited influence on clinical decision-making; however, results should be interpreted as observational associations rather than evidence of causal effects.
